# New Insights into Intrinsic Point Defects in V_2_VI_3_ Thermoelectric Materials

**DOI:** 10.1002/advs.201600004

**Published:** 2016-03-23

**Authors:** Tiejun Zhu, Lipeng Hu, Xinbing Zhao, Jian He

**Affiliations:** ^1^ State Key Laboratory of Silicon MaterialsSchool of Materials Science and EngineeringZhejiang UniversityHangzhou310027P.R. China; ^2^ Department of Physics and AstronomyClemson UniversityClemsonSC29634USA

**Keywords:** intrinsic point defects, V_2_VI_3_ semiconductors, Bi_2_Te_3_, thermoelectric properties, defect engineering

## Abstract

Defects and defect engineering are at the core of many regimes of material research, including the field of thermoelectric study. The 60‐year history of V_2_VI_3_ thermoelectric materials is a prime example of how a class of semiconductor material, considered mature several times, can be rejuvenated by better understanding and manipulation of defects. This review aims to provide a systematic account of the *underexplored* intrinsic point defects in V_2_VI_3_ compounds, with regard to (i) their formation and control, and (ii) their interplay with other types of defects towards higher thermoelectric performance. We herein present a convincing case that intrinsic point defects can be actively controlled by extrinsic doping and also via compositional, mechanical, and thermal control at various stages of material synthesis. An up‐to‐date understanding of intrinsic point defects in V_2_VI_3_ compounds is summarized in a (χ, r)‐model and applied to elucidating the donor‐like effect. These new insights not only enable more innovative defect engineering in other thermoelectric materials but also, in a broad context, contribute to rational defect design in advanced functional materials at large.

## 1 Introduction

V_2_VI_3_ compounds (V = Group V elements Sb and Bi, and VI = Group VI elements S, Se and Te) and their derivatives constitute an important class of semiconductor material in renewable energy and next generation information technology. For decades these compounds have been the benchmark thermoelectric (TE) materials.^1^ Recently they became a focus in the study of bulk quantum topological insulators.^2^ In this review we intend to address the fundamental yet underexplored role of *intrinsic point defects* in V_2_VI_3_ compounds. While this gap of knowledge has led to ambiguities in the synthesis‐structure‐property correlation, it is the area of research that holds the promise of further improving the material performance.

In V_2_VI_3_ materials intrinsic point defects specifically refer to vacancies and antisite defects while extrinsic point defects refer to guest atoms. Strictly speaking, intrinsic point defects are thermally created in an otherwise perfect crystal in which case the stoichiometry is retained. In practice, the condition of stoichiometry is often relaxed, so the nomenclature “intrinsic point defects” and “native point defects” can be used interchangeably. One should *not* confuse the nature of a point defect (i.e., intrinsic or extrinsic) with the character of the procedure of creating such defects (e.g., equilibrium or off‐equilibrium).

This review is written from *a thermoelectric perspective* in that we study the TE properties of the material in their totality to characterize intrinsic point defects and their interplay with other defects in V_2_VI_3_ materials; *reversely*, a better understanding of intrinsic point defects and their interplay with other defects enables us to further optimize the TE performance of V_2_VI_3_ compounds. The TE performance of a material is gauged by its dimensionless figure of merit *zT = σα* ^2^ *T*/*κ* = *PF*/*κ*,^3^ where *α*, *σ*,*κ*, *Τ* and *PF* are the Seebeck coefficient (i.e., thermopower), electrical conductivity, total thermal conductivity (including the lattice component, *κ*_ph_, and carrier component, *κ*_el_), the absolute temperature, and the power factor (*PF*), respectively. The overarching goal of TE research is to develop higher *zT* materials.^4^, ^5^, ^6^, ^7^, ^8^ To this end, *no* TE material would have achieved its best performance *without* defects.^9^ The canonical “phonon‐glass electron‐crystal” strategy is implemented via concertedly engineering point defects, textures, grain boundaries, and nanoinclusions to reduce the *κ*_ph_ 10, 11, 12, 13, 14, 15, 16, 17 and enhance the *PF*.^18^, ^19^, ^20^, ^21^, ^22^, ^23^, ^24^, ^25^

Bi_2_Te_3_ is customarily regarded as the representative of V_2_VI_3_ compounds. The crystal structure of this compound is trigonal (space group *R*‐3m) and consists of atomic layers stacked in the order of Te^(1)^–Bi–Te^(2)^–Bi–Te^(1)^ along the *c* axis. The Te atoms in site (2) have Te^(2)^–Bi bonds that are covalent and ionic, while the Te^(1)^–Te^(1)^ layers are weakly bound with van der Waals force. This conveys to the Bi_2_Te_3_ an almost two‐dimensional nature, with a strong anisotropy between the properties in the plane and those along the *c*‐axis. The pioneer work in Bi_2_Te_3_ by Goldsmid et al.,^26^ the first TE refrigerator using *p*‐ and n‐type Bi_2_Te_3_,^27^ and the classic alloying (solid solution) approach by Ioffe et al.^28^ dated back to 1950's. The outstanding TE performance and the wide range of composition in both p‐type and n‐type attained by alloying Bi_2_Te_3_ with isostructural Sb_2_Te_3_ or Bi_2_Se_3_ showcases the efficacy of defect engineering.^29^, ^30^, ^31^ In addition, V_2_VI_3_ compounds have been a test bed for novel material fabrication methods.^10^ These methods, in turn, govern *the type, amount, and topology* of defects. The history of TE study of V_2_VI_3_ compounds is a prime example of how a class of material can be rejuvenated by better understanding and manipulation of defects, ranging from 0D point defects,^32^ 1D dislocations,^33^, ^34^, ^35^ 2D grain boundaries,^36^, ^37^ to 3D nanoinclusions.^38^ This review focuses on intrinsic point defects in V_2_VI_3_ compounds.

Understanding the role of intrinsic point defects is a prerequisite for establishing the fundamental synthesis‐structure‐property correlation in V_2_VI_3_ semicoductors, however, it is challenging to correlate specific atomic level defects and macroscopic TE properties given the complex crystal structure, especially when heavily doped and/or in the presence of other types of defects. For example, the best commercial TE materials near room temperature are ternary p‐type Bi_2–_ *_x_*Sb*_x_*Te_3_ and n‐type Bi_2_Te_3–_ *_x_*Se*_x_*.^27^ The TE properties of these heavily *extrinsically* doped Bi_2_Te_3_ are actually governed by *intrinsic point defects*, the causal chain follows: extrinsic point defects → intrinsic point defects → TE properties (cf. Section 3.1).

The significance of intrinsic point defects in V_2_VI_3_ compounds is justified by a simple argument. The carrier concentration *n* is the material parameter of utmost importance in V_2_VI_3_ compounds as the *σ*, *α*, and *κ*_el_ depend closely on the value of *n*, intrinsic point defects are on a par with extrinsic point defects in the capacity of contributing charge carriers. Experimental and theoretical studies corroborated that the optimal carrier concentration *n* of V_2_VI_3_ materials is on the order of 10^19^ cm^–3^ 32, 33, 39 while intrinsic point defects alone can contribute 10^18^–10^20^ cm^–3^.^40^ In a proof‐of‐principle study, we devised an *intrinsic point defect engineering* (IPDE) approach in n‐type Bi_2_Te_2.3_Se_0.7_, which simultaneously optimized the *PF*, *κ*_ph_ and *zT*.^32^ The success of the IPDE approach is reflected in the *zT* peak value of above 1.2 at 445 K and also high averaged *zT* values of 1.1 between 300 K and 500 K.

The significance of intrinsic point defects is also confirmed in other state‐of‐the‐art TE materials. For example, vacancies modulate the carrier concentration *n* in Zintl compounds.^40^ In Mg_2_Si_0.4_Sn_0.6–_ *_x_*Sb*_x_*,^41^ Sb doping at low ratios tunes the *n* while it facilitates the formation of Mg vacancies at high Sb doping ratios, Mg vacancies effectively scatter heat‐carrying phonons; in addition, excess Mg in the starting material facilitates the formation of Mg interstitials that also alter the *n*. In ZrNiSn‐based half‐Heusler (HH) materials, the band gap is modulated by the content of Zr/Sn antisite defects.^42^

The rest of the article is organized as follows. We discuss the creation and control of intrinisc point defects in V_2_VI_3_ semiconductors in Section 2 and 3, respectively. As shown, intrinsic point defects can be created and manipulated compositionally, mechanically (via “the donor‐like effect”), and thermally (via “the recovery effect”). We propose a simple (*χ*, *r*)‐model and discuss the donor‐like effect. Section 4 and 5 are devoted to the impact of intrinsic point defects on the TE properties and how to engineer intrinsic point defects to tailor the material performance in different temperature ranges, respectively. In Section 6, we address the underheeded role of intrinsic point defects in nanostructuring and texturing process. Finally we conclude, in Section 7, with perspective remarks.

## 2 Formation of Intrinsic Point Defects in V_2_VI_3_ Binary Compounds

**Figure 1:**
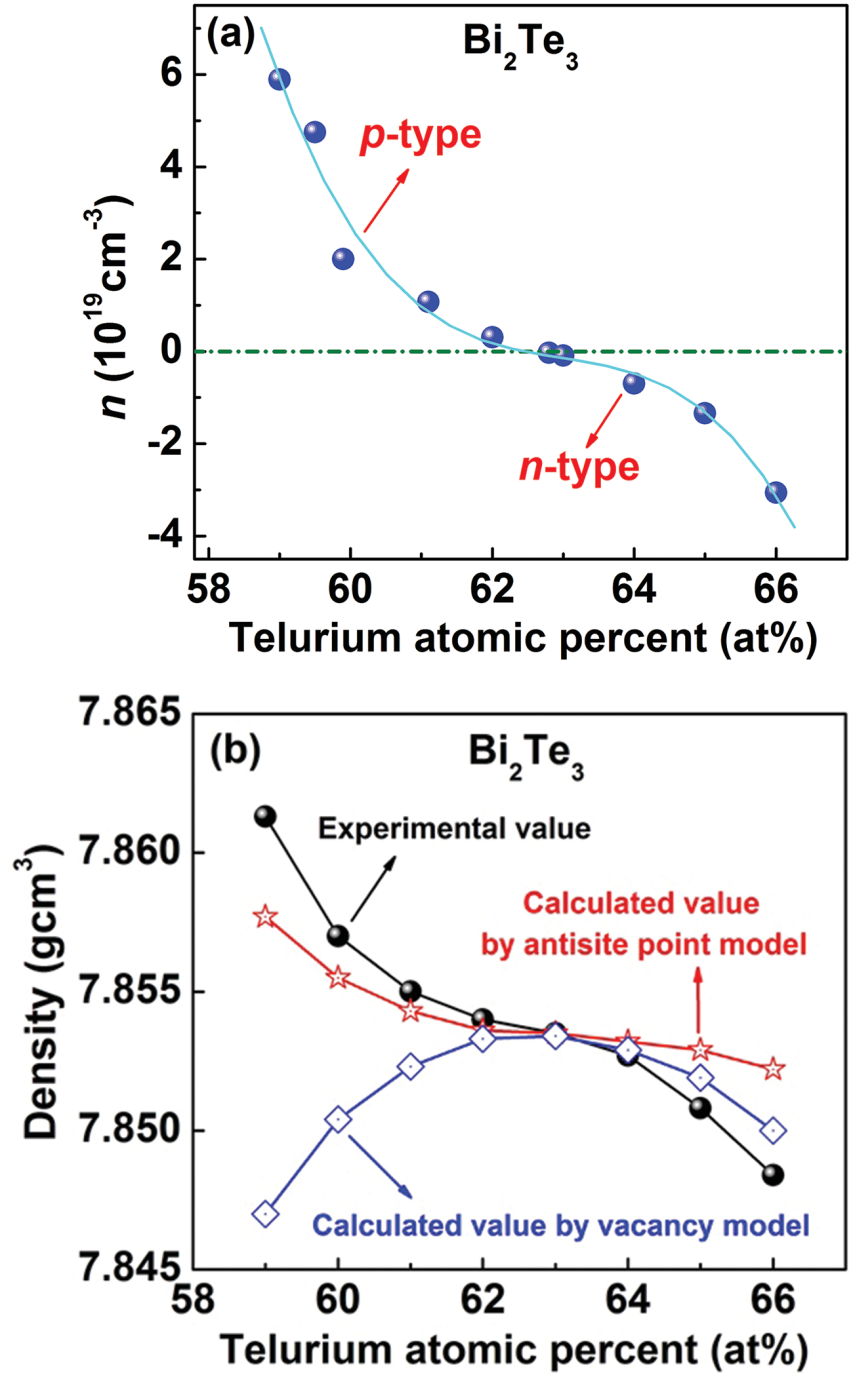
a) Room temperature carrier concentration of Bi_2_Te_3_ ingots as a function of Te content.^45^ b) Room temperature mass density of Bi_2_Te_3_ ingots as a function of Te content.^47^

The V_2_VI_3_ binary compounds grown from stoichiometric melts tend to have Group V element excess because Group VI element often precipitates as a secondary phase (mainly Te)^43^, ^44^ or is volatile (mainly S or Se).^32^ Satterthwaite et al. reported that the as‐grown Bi_2_Te_3_ ingot is p‐type when the actual Te content is less than 62.8 at% (namely, Te‐deficient), and the hole concentration *n*_h_ rapidly decreases with Te excess; at the other end, the ingot exhibits n‐type (**Figure** **1**a).^45^ These less intuitive observations can be explained by intrinsic point defects. Harman et al. proposed that the dominant intrinsic point defects in the as‐grown Bi_2_Te_3_ ingot are negatively charged antisite defects $\mathrm{Bi}\prime^{}{}_{\mathrm{Te}}$ on the Te‐deficient side and positively charged antisite defects ${\mathrm{Te}}_{\mathrm{Bi}}^{\cdot }$ on the Te‐rich side.^46^ This scenario is supported by the results of packing density measurements and the density values calculated for various defect models (Figure 1b).^47^ The presence of antisite defects in Bi_2_Te_3_ thin film is confirmed by high precision chemical analysis.^48^ In the same vein, the dominant intrinsic point defects in p‐type Sb_2_Te_3_ are $\mathrm{Sb}\prime^{}{}_{\mathrm{Te}}$,^49^, ^50^ while $V_{\mathrm{Se}}^{\cdot \cdot }$ are the dominant point defects in n‐type Bi_2_Se_3_.^51^, ^52^, ^53^ Horak et al. pointed out that $\mathrm{Bi}\prime^{}{}_{\mathrm{Se}}$ coexists with $V_{\mathrm{Se}}^{\cdot \cdot }$ in n‐type Bi_2_Se_3_.^53^ The predominance of these intrinsic point defects has been confirmed by first‐principles calculations.^54^, ^55^, ^56^, ^57^, ^58^, ^59^, ^60^ **Table** **1** lists the type and the concentration of dominant intrinsic point defects in Sb_2_Te_3_, Bi_2_Te_3_, and Bi_2_Se_3_.^58^

**Table 1:** Conduction type and the calculated concentration of dominant intrinsic point defect in Sb_2_Te_3_, Bi_2_Te_3_, and Bi_2_Se_3_. The concentration of defect depends on the formation energy and thus the position of Fermi level, which is fixed at the midgap in calculations.^58^

Compounds	Sb_2_Te_3_	Bi_2_Te_3_,	Bi_2_Se_3_
Conduction type	p	p	n
Point defect type	$\mathrm{Sb}\prime_{\mathrm{Te}}$	$\mathrm{Bi}\prime^{}{}_{\mathrm{Te}}$	$V_{\mathrm{Se}}^{\cdot \cdot }$
Point defect concentration (cm^–3^)	2 × 10^21^	8 × 10^19^	3 × 10^19^

In addition to intrinsic point defects, Bi excess (Te deficiency) may create extended defects such as the seven‐layer‐lamella defect ${\mathrm{Bi}}_{3}{\mathrm{Te}}_{4}^{\prime }$ with the sequence Te^1^–Bi–Te^2^–Bi–Te^2^–Bi–Te^1^. The presence of ${\mathrm{Bi}}_{3}{\mathrm{Te}}_{4}^{\prime }$ is confirmed by high resolution electron microscopy measurements in bulk crystals,^61^, ^62^ films,^63^ and nanowires of Bi_2_Te_3_.^64^ First‐principles calculations suggest that the low formation energy of the nearest neighbor X_Bi–Te1_ (i.e., the exchange of a Bi atom with a Te^1^ atom in the same supercell) facilitates the formation of ${\mathrm{Bi}}_{3}{\mathrm{Te}}_{4}^{\prime }$.^56^ Furthermore, Horak et al. proposed that $\mathrm{Bi}\prime_{\mathrm{Te}}$ and ${\mathrm{Bi}}_{3}{\mathrm{Te}}_{4}^{\prime }$ are favored at low and high Bi excess, respectively.^65^

## 3 Manipulation of Intrinsic Point Defects

In Section 3, we address how to implement intrinsic point defects in V_2_VI_3_ compounds, following the order of pre‐synthesis control (Section 3.1), in‐synthesis control (Section 3.2), and post‐synthesis control (Section 3.3 and Section 3.4). Alternatively, these controls can be categorized into compositional/chemical control (Section 3.1 and 3.2), mechanical control (Section 3.3), and thermal control (Section 3.4).

### 3.1 Compositional Control in Cation‐Rich V_2_VI_3_ Compounds

**Figure 2:**
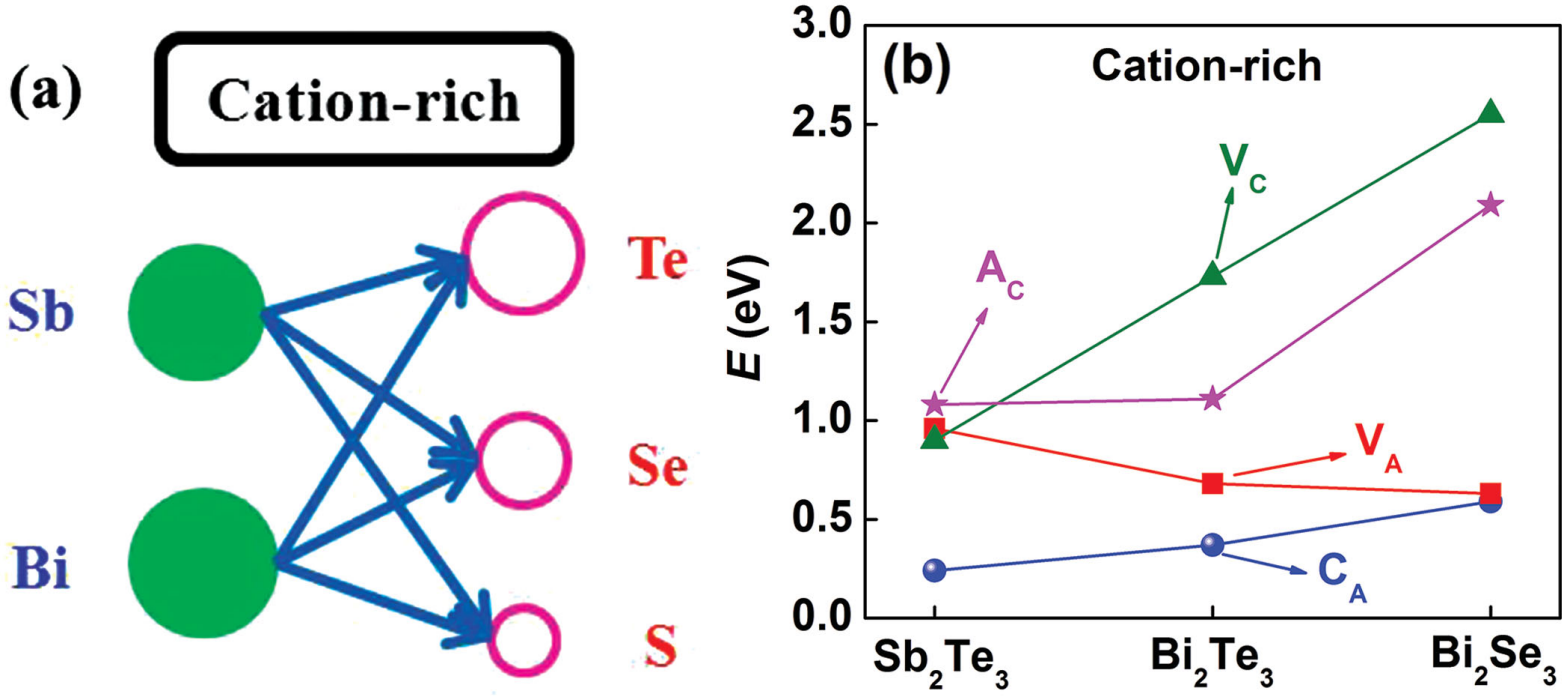
a) Schematic of the formation of intrinsic point defects in cation‐rich case. The size of circle represents the covalent radius. b) The calculated formation energies of intrinsic point defect considering spin‐orbit interactions in the cation‐rich case.^58^ V_C_, A_C_, V_A_, and C_A_ represent cation vacancies, anion antisite defects, anion vacancies, and cation antisite defects, respectively.

As mentioned in Section 2, V_2_VI_3_ *binary* compounds synthesized from stoichiometric starting composition tend to be cation rich. There is an important correlation between the conduction type and the carrier concentration of intrinsic point defects and the electronegativity *χ* and covalent radius *r* of cations and anions.^2^, ^32^, ^66^, ^67^, ^68^, ^69^, ^70^, ^71^ We hereafter call this correlation the *(χ, r)‐*mechanism or the *(χ, r)‐*model. As shown in **Figure** **2**a, the smaller the difference in *χ* and *r* between the cation and the anion the easier for antisite defects to form. At the other end, increasing the difference in *χ* and *r* between the cation and the anion will favor the formation of anion vacancies. We present the *χ*, *r* of constituent elements in binary V_2_VI_3_ compounds in **Table** **2**.

**Table 2:** Boiling point, electronegativity, covalent radius, and atomic mass of constituent elements in V_2_VI_3_ compounds

Elements	Bi	Sb	Te	Se	S
Boiling point [K]	1837	1860	1261	1231	991
Electronegativity	2.02	2.05	2.1	2.55	2.58
Covalent radius [Å]	1.46	1.40	1.36	1.16	1.02
Atomic mass	208.98	121.75	127.60	78.96	32.07

The formation energy of antisite defect E_AS_ can be enumerated in ascending order as:

(1) $$\begin{array}{l} E_{\mathrm{AS}}\left(\mathrm{Sb–Te}\right)<E_{\mathrm{AS}}\left(\mathrm{Bi–Te}\right)<E_{\mathrm{AS}}\left(\mathrm{Sb–Se}\right)\\ \;\;\;<E_{\mathrm{AS}}\left(\mathrm{Bi–Se}\right)<E_{\mathrm{AS}}\left(\mathrm{Bi–S}\right) \end{array}$$

Meanwhile, the formation energy of anion vacancies *E*_V_ is listed in descending order as:

(2) $$\begin{array}{l} E_{V}\left(\mathrm{Sb–Te}\right)>E_{V}\left(\mathrm{Bi–Te}\right)>E_{V}\left(\mathrm{Sb–Se}\right)\\ \;\;\;>E_{V}\left(\mathrm{Bi–Se}\right)>E_{V}\left(\mathrm{Bi–S}\right) \end{array}$$

These two inequalities can be used to *semi‐quantitatively* explain the composition dependence of the conduction type and that of the carrier concentration in cation‐rich V_2_VI_3_ single crystals and zone‐melted ingots.^32^ For example, the difference in *E_AS_* explains why Sb_2_Te_3_ exhibits a strong p‐type characteristic whereas Bi_2_Te_3_ is weakly p‐type in light of inequality (1). Inequality (2) can explain the strong n‐type characteristic of Bi_2_Se_3_ in terms of the low E_V_.

The formation energy of $\mathrm{Bi}\prime^{}{}_{\mathrm{Te}}$ in cation‐rich binary Bi_2_Te_3_ can be calculated by the following formula derived from statistical thermodynamics:^47^

(3) $$E_{B_{\mathrm{Te}}^{\prime }}=-k_{b}T_{m}\left(\ln\frac{n_{B_{\mathrm{Te}}^{\prime }}}{N_{\mathrm{Te}}}+1\right)$$

where *k*_b_ is the Boltzmann constant, *T*_m_ the melting point, $n_{B_{\mathrm{Te}}^{\prime }}$ the number of $B_{\mathrm{Te}}^{\prime }$ per cm^3^, and $N_{\mathrm{Te}}$ the number of available Te sites per cm^3^, respectively. The typical value of *E*_AS_ is 0.35 eV, 0.50 eV, 0.64 eV for binary Sb_2_Te_3_, Bi_2_Te_3_, and Bi_2_Se_3_, respectively.^47^, ^49^, ^50^, ^53^ These values are confirmed by first‐principles calculations (Figure 2b). Generally, cation antisite defects and anion vacancies are energetically more favorable than anion antisite defects and cation vacancies under the cation‐rich condition.^58^

**Figure 3:**
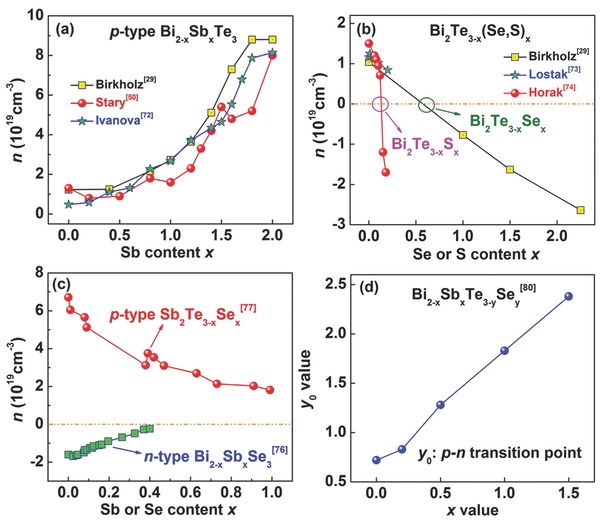
a) Room temperature carrier concentration of unidirectionally grown p‐type Bi_2–_ *_x_*Sb*_x_*Te_3_ as a function of Sb content.^29^, ^50^, ^72^ b) Room temperature carrier concentration of unidirectionally grown n‐type Bi_2_Te_3–*x*_(Se/S)_*x*_ as a function of Se or S content.^29^, ^73^, ^74^ c) Room temperature carrier concentration of unidirectionally grown p‐type Sb_2_Te_3–*x*_Se_3_ and p‐type Bi_2–*x*_Sb_x_Se_3_ as a function of Se content and Sb content, respectively.^76^, ^77^ d) The *y*_0_ value (the Sb content at which the p–n transition occurs) of unidirectionally grown Bi_2–*y*_Sb_*y*_Te_3–*x*_Se_*x*_ as a function of Se content *x*.^80^

The (*χ*, *r*)‐model can be extended to *ternary* and *quaternary* V_2_VI_3_ materials. In general, substituting more electronegative or smaller atoms of the same valence on the cation site tends to drive the material towards hole‐like (p‐type) conduction, while substituting more electronegative or smaller atoms of the same valence on the anion site tends to drive the system towards electron‐like (n‐type) conduction. For instance, increasing Sb content in p‐type Bi_2–_ *_x_*Sb*_x_*Te_3_ reduces the E_AS_, thereby increasing the hole concentration *n*_h_ owing to a smaller difference in *χ* and *r* between Sb and Te than the counterpart between Bi and Te (**Figure** **3**a).^29^, ^50^, ^72^ Similarly, substituting Te by Se in Bi_2_Te_3_ increases the E_AS_ and supresses the E_V_, resulting in a n‐type conduction.^32^ When the concentration of anion vacancies ($\left[V_{\mathrm{Te}}^{\cdot \cdot }\right]$ and $\left[V_{\mathrm{Se}}^{\cdot \cdot }\right]$) exceeds the concentration of antisite defects ($\left[\mathrm{Bi}\prime^{}{}_{\mathrm{Te}}\right]$ and $\left[{\mathrm{Bi}}^{\prime }{}_{\mathrm{Se}}\right]$), a p‐type to n‐type crossover occurs (Figure 3b).^32^, ^73^ Notably, S substitution on the Se‐site quickly shifts the p–n crossover point down to *x* = 0.13.^68^, ^74^, ^75^ Doping n‐type Bi_2_Se_3_ with Sb^76^ or doping p‐type Sb_2_Te_3_ with Se^77^, ^78^, ^79^ rapidly diminish the carrier concentration (Figure 3c). Teramoto et al. found that *y*_0_, the *y* value at which the p–n transition occurs, increases with the *x* value in the Sb*_x_*Bi_2–_ *_x_*Te_3–_ *_y_*Se*_y_* quaternary system (Figure 3d).^80^

An important implication of these results is that *intrinsic point defects* can be actively tuned by *isoelectron extrinsic dopants*. The best commercial room temperature TE materials p‐type Bi_2–_ *_x_*Sb*_x_*Te_3_ and n‐type Bi_2_Te_3–_ *_x_*Se*_x_* alloys are good examples. These results also serve as a caveat when we try to derive the causal chain in data analysis: implementing isoelectron extrinsic dopants leads to the formation of intrisinsic point defects, then the latter govern the observed conduction type and the magnitude of carrier concentration.

Comparing to the case of *isoelectron extrinsic doping*, the interplay between *intrinsic point defects* and *heteroelectron extrinsic dopants* is more complex. Nonetheless, it is known that *heteroelectron* dopants such as Li,^81^ Ag,^82^, ^83^ Cu,^84^ Pb,^85^, ^86^, ^87^ Sn,^88^ I,^89^ Mn,^90^ Ge,^91^ affect the formation of intrinsic point defects. For example, Te loss can be suppressed by adding a small amount of Cu to increase the formation energy of $V_{\mathrm{Te}}^{\cdot \cdot }$ 92

**Figure 4:**
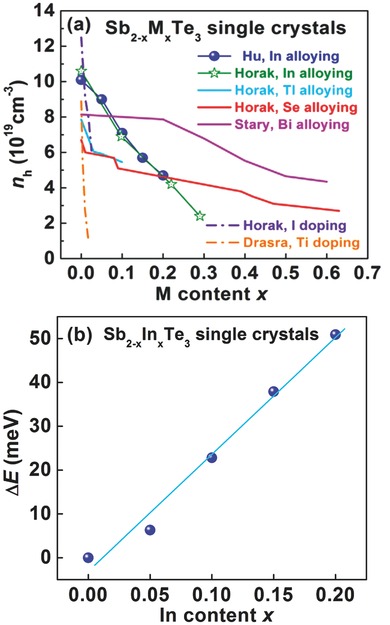
a) Composition dependence of the hole concentration of Sb_2–_ *_x_*M*_x_*Te_3_ single crystals.^94^ b) Indium content dependence of formation energy of $\mathrm{Sb}\prime_{\mathrm{Te}}$ in Sb_2–_ *_x_*In*_x_*Te_3_ single crystals.^94^ Reproduced with permission.^94^ Copyright 2015, Elsevier.

Indium (In) doping is a manifestation of the significance of intrinsic point defects in the presence of *heteroelectron* extrinsic dopants. Doping by indium modulates the formation energy of intrinsic point defects and thus alters the carrier concentration, shifting the optimal operation regime from room temperature to higher temperature.^93^, ^94^ Indium (5s^2^5p^1^) occupying the Sb (5s^2^5p^3^)‐site is expected to form a negatively charged substitutional point defect and thus increases the hole concentration *n*_h_ ($\mathrm{In}\Rightarrow {\mathrm{In}}_{\mathrm{Sb}}^{\prime \prime }+2h^{•}$). However, **Figure** **4**a shows the opposite: indium substitution moderately decreases the *n*_h_ in Sb_2–_ *_x_*In*_x_*Te_3_,^94^ in contrast to iodine doping^95^ and Ti doping.^96^

To explain this counter‐intuitive observation, Horakproposed that incorporation of indium into Sb_2_Te_3_ creates charge neutral point defects ${\mathrm{In}}_{\mathrm{Sb}}^{\times }$, accompanied by a $\mathrm{In}({\mathrm{5s}}^{2}{\mathrm{5p}}^{1})\to {\mathrm{In}}_{\mathrm{Sb}}^{x}(5s^{0}5p^{3})$ electronic transition.^71^ In this scenario, the substitution of Sb by In does *not* directly alter the *n*_h_, rather, it raises the *E*_AS_ due to the greater difference in *χ* between In and Te than that between Sb and Te, thereby reducing the *n*_h_. A similar scenario has been proposed for Tl,^97^ Bi,^50^ or Se^77^‐doped Sb_2_Te_3_. The relative reduction of *n*_h_ due to the doping on the Te‐site is enumerated in descending order as: Tl > In > Se > Bi.^94^

To elucidate the effect of indium doping on the *n*_h_, the *E*_AS_ of $\mathrm{Sb}\prime_{\mathrm{Te}}$ is estimated by the following relation:^94^

(4) $$N_{\mathrm{AS}}\propto (C_{\mathrm{Sb}}-C_{\mathrm{In}})\;\exp\left(-\frac{E_{\mathrm{AS}}}{k_{b}T_{m}}\right)$$

where *N*_AS_ is the concentration of antisite, *C*_Sb_ the Sb concentration, *C*_In_ the In concentration, *k*_b_ the Boltzmann constant, *T*_m_ the melting point (assuming a linear relationship between *T*_m_(Sb_2_Te_3_) = 902 K and *T*_m_(In_2_Te_3_) = 940 K), respectively. *E*_AS_ = *E*_0_ + Δ*E*, where *E*_0_ ≈ 0.35 eV is the activation energy of $\mathrm{Sb}\prime_{\mathrm{Te}}$ in undoped Sb_2_Te_3_,^49^ and Δ*E* the activation energy increment of $\mathrm{Sb}\prime_{\mathrm{Te}}$ due to indium doping. As shown in Figure 4b, the Δ*E* rapidly increases with increasing indium content, a reflection of the fact that the formation energy of $\mathrm{Sb}\prime_{\mathrm{Te}}$ in indium‐doped Sb_2_Te_3_ is higher than that of undoped one.^94^

### 3.2 Synthesis Environment Control

**Figure 5:**
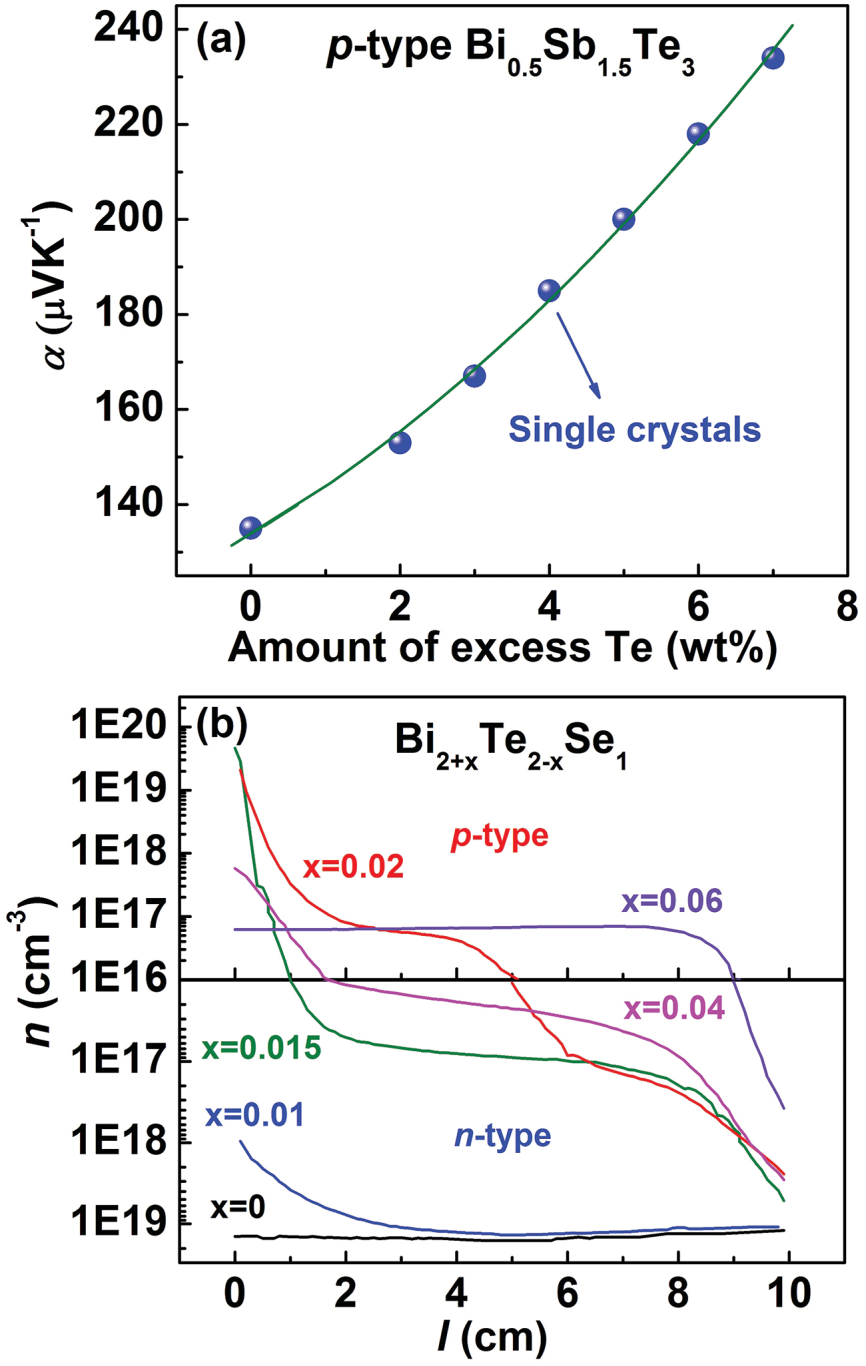
a) Room temperature Seebeck coefficient of p‐type Bi_0.5_Sb_1.5_Te_3_ single crystals as a function of excess Te content.^43^ b) Carrier concentration measured at *T* = 10 K of Bi_2+_ *_x_*Te_2−_ *_x_*Se with *x* = 0, 0.015, 0.02, 0.04, and 0.06 at different positions in the crystal boule (“0” marks the end of the crystal boule).^99^

We in Section 3.2 discuss the control of intrinsic point defects in the case of off‐stoichiometric starting materials. We hereafter call this mechanism “synthesis environment control”. Under a cation‐rich growth condition, $V_{\mathrm{Se}}^{\cdot \cdot }$, $\mathrm{Bi}\prime_{\mathrm{Te}}$, and $\mathrm{Sb}\prime_{\mathrm{Te}}$ are responsible for the native n‐, p‐, and p‐type conduction in Bi_2_Se_3_, Bi_2_Te_3_, and Sb_2_Te_3_ ingots, respectively. Under an anion‐rich condition, ${\mathrm{Se}}_{\mathrm{Bi}}^{\cdot }$, ${\mathrm{Te}}_{\mathrm{Bi}}^{\cdot }$, and $V\prime \prime \prime \prime_{\mathrm{Sb}}$ are responsible for the native n‐type, n‐type, and p‐type conduction in Bi_2_Se_3_, Bi_2_Te_3_, and Sb_2_Te_3_ ingots, respectively.^58^, ^98^ In the zone‐melted (ZM) p‐type Bi_0.5_Sb_1.5_Te_3_ ingots, it is found that the formation of antisite defects can be suppressed by adding extra Te (>60 at%) to the melts because the *E*_AS_ is higher under a Te‐rich condition (**Figure** **5**a).^27^, ^43^ Meanwhile, excess Bi (>40 at%) in Bi_2_Te_3_ and Bi_2_Se_3_ facilitates the formation of $\mathrm{Bi}\prime_{\mathrm{Te}}$ and $\mathrm{Bi}\prime_{\mathrm{Se}}$. Horak et al. pointed out that the p‐type ZM B_2+_ *_x_*Te_3_ and n‐type ZM B_2+_ *_x_*Se_3_ ingots show an increase of the *n*_h_ and a decrease of *n*_e_ because of the increase of $\mathrm{Bi}\prime_{\mathrm{Te}}$ and $\mathrm{Bi}\prime_{\mathrm{Se}}$ concentration with increasing *x*, respectively.^65^

Controlling intrinsic point defects in V_2_VI_3_ compounds now has an impact beyond the field of TE material research. Bi_2_Te_2_Se becomes the subject of crystal growth research owing to its topological insulator properties.^2^, ^29^, ^32^ In the study of 3D topological insulators a grand challenge is to minimize the bulk electrical conduction to help discern the surface electrical conduction. However, stoichiometric Bi_2_Te_2_Se grown by a modified Bridgman method is metal‐like, with a *n*_e_ on the order of 10^19^ cm^–3^. To suppress the bulk electrical conduction, Jia et al. fabricated highly bulk resistive Bi_2+_ *_x_*Te_2−_ *_x_*Se samples under a slightly Bi‐rich condition in which the Bi excess introduces $\mathrm{Bi}\prime_{\mathrm{Te}}$ (Figure 5b).^99^ In another study of topological insulator, Jiang et al. fabricated high‐quality Sb_2_Te_3_ films by molecular beam epitaxy and observed the intrinsic point defects by in situ scanning tunneling microscopy and spectroscopy. They found that in a strong Te‐rich environment $V\prime \prime \prime \prime_{\mathrm{Sb}}$ is the defect with lowest formation energy while $\mathrm{Sb}\prime_{\mathrm{Te}}$ becomes the lowest energy defect in a less Te‐rich environment.^100^

### 3.3 Mechanical Control: Deformation and the Donor‐like Effect

**Figure 6:**
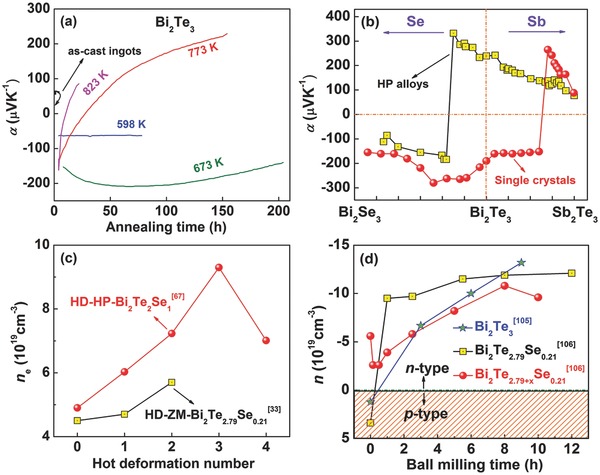
a) Room temperature Seebeck coefficient of extruded Bi_2_Te_3_ingot as a function of annealing time at different annealing temperatures.^101^ b) Room temperature Seebeck coefficient of (Bi,Sb)_2_(Te,Se)_3_ single crystals and HP alloys as a function of Se and Sb doping ratios.^103^ c) Room temperature carrier concentration of Bi_2_Te_3–_ *_x_*Se*_x_* alloys as a function of the number of times of hot deformation.^33^, ^67^ d) Room temperature carrier concentration of Bi_2_Te_3–_ *_x_*Se*_x_* alloys as a function of ball milling time.^105^, ^106^

In addition to compositional control (Section 3.1) and synthesis environment control (Section 3.2), mechanical control via post‐synthesis deformation is another approach.^101^, ^102^, ^103^, ^104^, ^105^, ^106^, ^107^, ^108^ It is well known that the p‐type Bi_2_Te_3_ ingots can be inverted to n‐type simply by pressing,^109^ and the pressed n‐type material can be *re‐inverted* to p‐type via sintering at sufficiently high temperatures.^110^ Heavy plastic deformation of Bi_2_Te_3_ ingots produces non‐basal slips and $V_{\mathrm{Te}}^{\cdot \cdot }$, changing the conduction type from p‐type to n‐type, and simultaneously enhancing the electrical conductivity (**Figure** **6**a).^101^ Ionescu et al. suggested that non‐basal slip produces 3 Bi to 2 Te vacancy–interstitial pairs during heavy deformation processing.^111^ In presence of Bi vacancies, Bi atoms diffuse from Te sites back to their original sublattice sites, *extra* Te vacancies and excess electrons are thus produced. This important mechanism is called “*the donor‐like effect*”, expressed as:

(5) $$2V_{\mathrm{Bi}}^{\prime \prime \prime }+3V_{\mathrm{Te}}^{••}+{\mathrm{Bi}}_{\mathrm{Te}}^{\prime }\Rightarrow V_{\mathrm{Bi}}^{\prime \prime \prime }+{\mathrm{Bi}}_{\mathrm{Bi}}^{x}+4V_{\mathrm{Te}}^{••}+6e^{\prime }$$

where $V_{\mathrm{Bi}}^{\prime \prime \prime }$ and $V_{\mathrm{Te}}^{\cdot \cdot }$ are the Bi and Te vacancies, $B_{\mathrm{Te}}^{\prime }$ the antisite defects, and $e^{\prime }$ the excess electrons, respectively. Similar formulae like (5) hold for $V\prime \prime \prime \prime_{\mathrm{Sb}}$, $\mathrm{Sb}\prime_{\mathrm{Te}}$, $V_{\mathrm{Se}}^{\cdot \cdot }$, and $\mathrm{Bi}\prime_{\mathrm{Se}}$.

The donor‐like effect is a delicate multiple‐stage n‐type doping mechanism involving multiple intrinsic point defects. The study of donor‐like effect is warranted because grinding, ball milling, hot/cold deformation, and hot pressing processes have been extensively used for various purposes in TE research. The past decade has witnessed great strides toward understanding and untilizing the donor‐like effect in V_2_VI_3_ compounds.

Experimentally, the impact of donor‐like effect is reflected in the large difference in *α*, which is inversely correlated to the *n*, between (Bi,Sb)_2_(Te,Se)_3_ single crystal and hot pressed (HP) sample (Figure 6b).^103^ The (Bi,Sb)_2_(Te,Se)_3_ single crystal shows a p‐type conduction for all compositions of Bi_2–_ *_x_*Sb*_x_*Te_3_ and a p–n transition at Bi_2_Te_2.1_Se_0.9_, in contrast, the HP sample exhibits a p–n transition at Bi_0.66_Sb_1.34_Te_3_ and a n‐type conduction for all compositions of Bi_2_Te_3–_ *_x_*Se*_x_*. The donor‐like effect can explain these observations. Importantly, hot deformation (HD) processes promote the donor‐like effect.^2^, ^32^, ^33^, ^66^, ^67^, ^112^ Different from the strong donor‐like effect created by heavy deformation such as grinding, ball milling (BM), and extrusion, HD produces weaker donor‐like effect due to milder deformation.

More severe deformation produces finer powders and a greater decrease in the *n*_h_ in p‐type Bi_2–_ *_x_*Sb*_x_*Te_3_ 113 and a larger increase in the *n*_e_ in n‐type Bi_2_Te_3−_ *_x_*Se*_x_*.^114^ Shin et al. deformed the p‐type Bi_0.5_Sb_1.5_Te_3_ ingot by cold pressing at 700 MPa from one to eleven times using the tool steel mold.^115^ They showed that the *α* increases with the increasing number of times of cold pressing, which correlates with a stronger donor‐like effect. Similarly, increasing the number of times of HD^33^, ^67^ or prolonging the BM time^105^, ^106^ facilitates the donor‐like effect and hence increased the *n*_e_ in n‐type Bi_2_Te_3−_ *_x_*Se*_x_* (Figure 6c and 6d). The donor‐like effect gets marginal above a certain level of deformation.

### 3.4 Thermal Control via the Recovery Effect

The recovery effect can be regarded as a *post‐deformation* thermal relaxation process, which basically counters the donor‐like effect regarding the carrier concentration change. The microscopic picture of the recovery effect posits that anion vacancies are annihilated by dislocation climb and array formation upon annealing. As expected, the recovery effect has a strong dependence on the annealing temperature (Figure 6a).^101^

Low‐temperature annealing only slightly mitigates the donor‐like effect and thus slightly reduces the *n*_e_. As a result, the *σ* is reduced while the *α* is somewhat enhanced. At the other end, when the deformed samples are annealed at high temperatures for a long time, the donor‐like effect can be nearly removed, as a result, the electrical properties revert slowly back to the original ones. Studies also showed that raising HP or SPS temperatures also mitigates the donor‐like effect (i.e., $V_{\mathrm{Te}}^{\cdot \cdot }$ or $V_{\mathrm{Se}}^{\cdot \cdot }$) due to the recovery effect.^32^, ^116^, ^117^, ^118^ It should be pointed out that the HD and annealing temperatrue are substantially higher than the operation temperature of V_2_VI_3_ TE materials so the thermal stability of as formed intrinsic point defects is *not* an issue in operation. This has been confirmed by our repetitive test measurements.

## 4 Role of Intrinsic Point Defect towards Higher *zT*

In Section 4 we address how intrinsic point defects generally impact the TE properties *σ*, *α* and *κ*, which sets the stage for elucidating intrinsic point defect engineering in Section 5.

### 4.1 Optimizing Electron Band Structure

Optimizing electron band structure involves two basic tasks: (i) tuning the *band filling* to attain an optimal carrier concentration *n*; and (ii) enhancing the electron density of states (DOS) near the Fermi level *E*_F_ to increase the *α*. While implementing extrinsic point defects by doping remains the mainstream methodology to optimize the value of *n*, we recently showed that intrinsic point defects alone can attain an optimal *n* value ≈ 5 × 10^19^ cm^–3^ in both p‐ and n‐type V_2_VI_3_ materials.^32^ To enhance the DOS near *E*_F_, theoretical calculations by Hashibon et al. showed that the *E_F_* is shifted into the valence band by $\mathrm{Bi}\prime_{\mathrm{Te}}$, and into the conduction band by ${\mathrm{Te}}_{\mathrm{Bi}}^{•}$,^56^ forming resonant (defect) states.^21^ On the other hand, the band structure tuning by intrinsic point defects In V_2_VI_3_ compounds will strongly interplay with composition optimization, which results in the change in band gap. The discussion on this topic is specially presented in Section 5.2.

### 4.2 Reduced Lattice Thermal Conductivity

**Figure 7:**
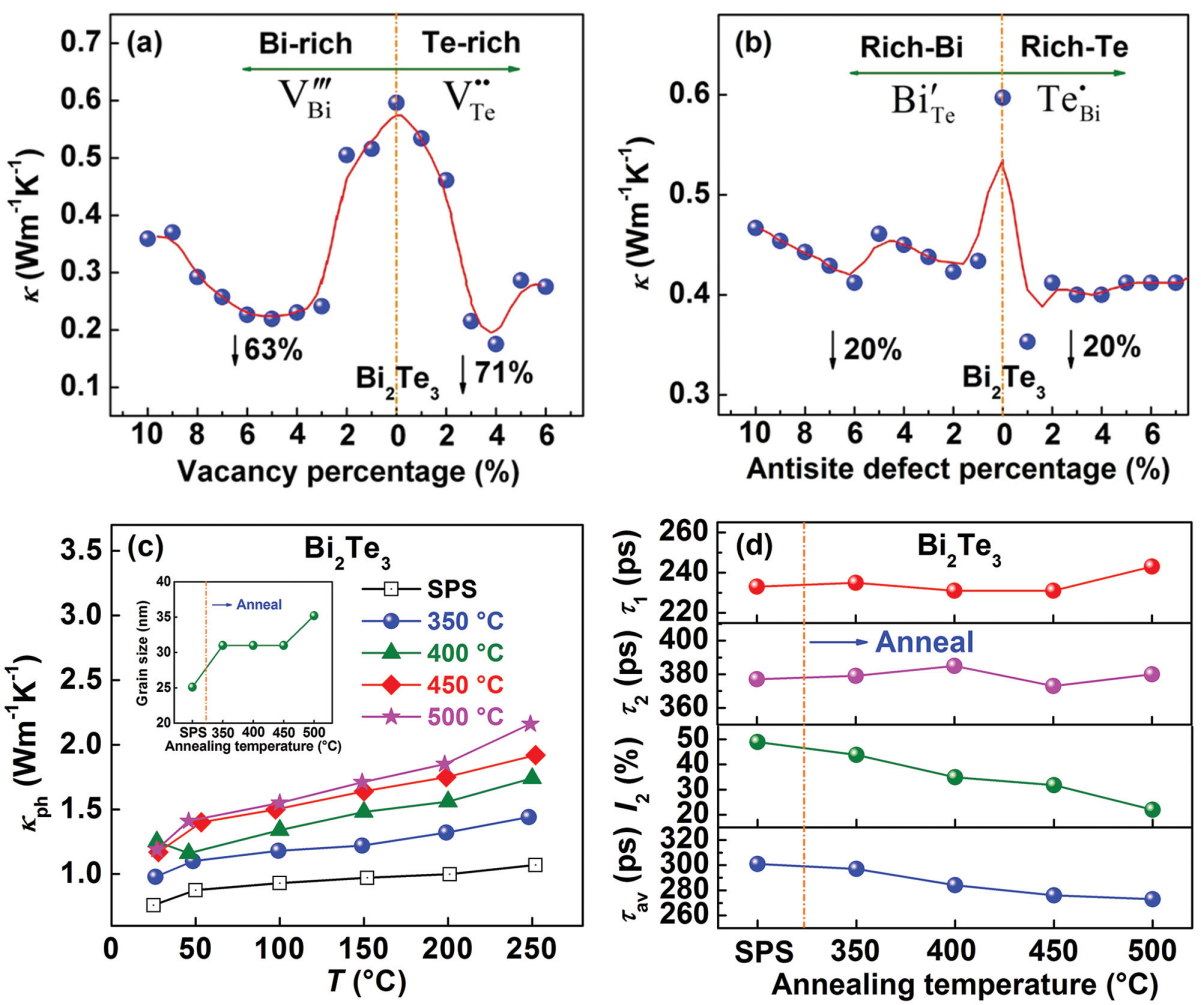
Calculated lattice thermal conductivity for defected bulk Bi_2_Te_3_ obtained from NEMD for a system size of 8 × 8 × 4 cells as a function of the a) vacancy defect percentage, and b) antisite defect percentage.^119^ c) Temperature dependent lattice thermal conductivity of Bi_2_Te_3_ nanocrystals annealed at different temperatures (The inset presents the grain size of Bi_2_Te_3_ nanocrystals annealed at different temperatures). d) Variations of positron lifetime *τ*_1_, *τ*_2_, intensity *I*_2_, and average lifetime *τ*_av_ as a function of annealing temperature.^120^

Compared to the closely inter‐dependent *σ*, *α*, *κ*_el_, the *κ*_ph_ is the only TE property that can be tuned fairly independently. To date, the basic strategy to reduce the *κ*_ph_ is to introduce more and diverse phonon scattering centers because heat‐carrying phonons have a wide distribution in energy (frequency) and momentum (wavelength). Intrinsic point defects are effective phonon scatters above room temperature because the average wavelength of heat‐carrying phonons gets shorter at elevated temperatures. Termentzidis et al. studied the effects of vacancies and antisite defects on the *κ*_ph_ by non‐equilibrium molecular dynamics simulations (NEMD).^119^ The reduction of *κ*_ph_ is >60% for 5 % [$V\prime \prime \prime \prime_{\mathrm{Bi}}$] and > 70% for 4 % [$V_{\mathrm{Te}}^{\cdot \cdot }$] in Bi_2_Te_3_ (**Figure** **7**a). In contrast, the reduction in *κ*_ph_ is about 20% regardless of the concentration of $\mathrm{Bi}\prime_{\mathrm{Te}}$ or ${\mathrm{Te}}_{\mathrm{Bi}}^{•}$ (Figure 7b). These results are understandable in that the vacancies possess larger mass difference and larger strain fluctuation than the antisite defects, thus more effectively scattering heat‐carrying phonons.

Grain boundaries provide us with another effective phonon scattering mechanism. At first glance, grain boundaries are irrelevant to intrinsic point defects. However, the routine powder metallurgy methods used to refine grain size often involve deformation processes that create vacancies (c.f. Section 3.3).^32^, ^120^ The specific contribution of vacancies to the reduction of *κ*_ph_ is often unaccounted or mistakenly attributed to grain boundaries. He et al. recently studied the relation between point defects, grain boundaries, and the reduction *κ*_ph_ in Bi_2_Te_3_ nanocrystals by means of thermal conductivity, electron microscopy, and positron annihilation measurements.^120^ It is instructive to note that the *κ*_ph_ of Bi_2_Te_3_ nanocrystals increases with an increasing annealing temperature but the grain size barely changes upon annealing (Figure 7c). Positron annihilation lifetime measurements indicated a gradual reduction of vacancy concentration upon annealing (Figure 7d). Hence the reduction of *κ*_ph_ in Bi_2_Te_3_ nanocrystals is due to phonon scattering by vacancies rather than grain boundaries.

## 5 Intrinsic Point Defect Engineering

In this section, we discuss how to engineer intrinsic point defect to optimize the material's TE performance in different temperature ranges. In view of the donor‐like effect and the recovery effect, it is imperative to compare the behavior of single crystal, ZM ingot, HP and HD sample in relation to their synthesis and deformation conditions. All the HP samples are prepared from ballmilled powder, if not otherwise noted.

### 5.1 Reassessment of Optimal Compositions

V_2_VI_3_‐based compounds are often subject to powder metallurgy processes such as BM, HP, and HD etc. The donor‐like effect (cf. Section 3.3) and the recovery effect (cf. Section 3.4) thus make the optimal composition of n‐ and p‐type HP and HD V_2_VI_3_ materials different from that of a single crystal or a ZM ingot.

#### 5.1.1 n‐type *Ternary Bi_2_Te_3_*_–_ *_x_Se_x_*

**Figure 8:**
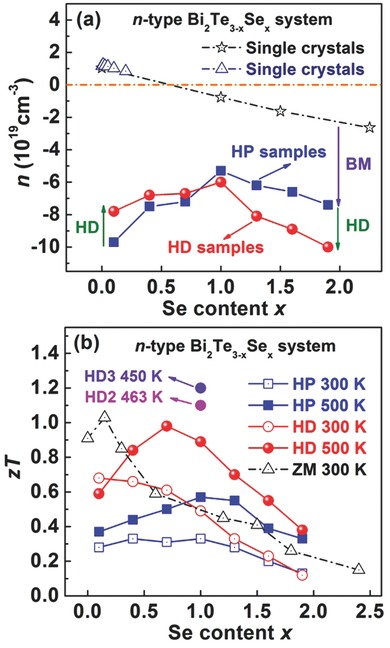
a) Room temperature carrier concentration of the undoped single crystals,^29^, ^73^ HP and HD polycrystalline^32^ Bi_2_Te_3–__x_Se*_x_* samples. The arrows are to help visualize the trend of carrier concentration variation upon BM and HD processing. b) Se content dependences of zT of the ZM,^39^ HP and HD^32^ Bi_2_Te_3–_ *_x_*Se*_x_* samples. All thermoelectric properties are measured along the in‐plane direction. Reproduced with permission.^32^

In light of the (*χ*, *r*)‐mechanism (cf. Section 2), and the greater difference in *χ* and *r* between Bi and Se than that between Bi and Te (cf. Table 2), substituting Te by Se in Bi_2_Te_3_ single crystal increases the E_AS_ and decreases the E_V,_ resulting in a p‐type conduction. A p–n crossover occurs when the electrons contributed by anion vacancies ($V_{\mathrm{Te}}^{\cdot \cdot }$ and $V_{\mathrm{Se}}^{\cdot \cdot }$) outnumber the holes created by antisite defects ($\mathrm{Bi}\prime_{\mathrm{Te}}$ and $\mathrm{Bi}\prime_{\mathrm{Se}}$) (**Figure** **8**a). Unidirectionally grown Bi_2_Te_3–_ *_x_*Se*_x_* ZM ingots have optimal compositions at *x* = 0.15–0.3, showing a weak p‐type conduction because of the predominance of $\mathrm{Bi}\prime_{\mathrm{Te}}$ and $\mathrm{Bi}\prime_{\mathrm{Se}}$.^29^, ^73^ Electron doping by halide inhibits the p–n crossover and attains an optimal electron concentration *n*_e_ ≈ 5 × 10^19^ cm^–3^.^32^, ^33^ Notably, inhibiting the p–n crossover can be achieved by the donor‐like effect.^101^, ^103^ Figure 8a shows that the donor‐like effect gives rise to a high *n*_e_ value, all the HP and HD samples are n‐type conductive, especially at the traditional optimal compositions *x* = 0.15–0.3.^32^

Figure 8 showcases the effects of compositional, mechanical, and thermal control of intrinsic point defects in n‐type ternary Bi_2_Te_3–_ *_x_*Se*_x_*. As shown in Figure 8a, the *n*_e_ value of the HD and HP sample firstly decreases and then increases with increasing Se content *x*. We first look at the HP sample. Increasing Se content in the synthesis stage suppresses the formation of antisite defects. Because the intensity of donor‐like effect is directly correlated with the concentration of antisite defects (Equation (5)), a reduced concentration of antisite defects leads to the reduced *n*_e_, which is the case at *x* < 1. The optimal composition for n‐type polycrystals is shifted to *x* = 0.7–1.0 with a *n*_e_ ≈ 5–7 × 10^19^ cm^–3^ (Figure 8a).^32^ To understand the change of slope of *n*_e_ at *x* >1 in the HP sample, one has to take into account the volatility of Se. The Se loss tends to be more severe at higher *x*, resulting in a higher concentration of antisite defects and thereby increasing the *n*_e_.

We may understand the behavior of the HD sample in a similar way, the only extra consideration is the recovery effect (cf. Section 3.4). Figure 8a shows that the HD sample has a *n*_e_ value consistently lower than the HP sample at *x* < 1.0, above which it is the opposite. We thus infer that nearly all antisite defects participate in the donor‐like effect (cf. Equation (5)) in the HD sample at *x* < 1.0, the recovery effect sets in and mitigates the donor‐like effect, leading to a lower value of n_e_.^32^, ^104^ At high Se contents (*x* > 1.0), however, there is a higher concentration of antisite defects due to the Se loss. As such, a portion of antisite defects participates in the donor‐like effect during the BM process, the remainder of antisite defects participate in the donor‐like effect during the HD process (Figure 8a).^32^ The stronger donor‐like effect gives rises to a higher *n*_e_ value (>6 × 10^19^ cm^–3^) than in the HP sample. Such a *n*_e_ value is favorable for a high *PF* but too high for a good *zT*.

Intrinsic point defects impact the *κ*_ph_ as well. Both antisite defects and vacancies reduce the *κ*_ph_, but the impact of vacancies is much greater because of the larger mass and size differences.^119^ The deformation‐induced vacancies $V\prime \prime \prime \prime_{\mathrm{Bi}}$ and $V_{\mathrm{Te}}^{\cdot \cdot }$ (or $V_{\mathrm{Se}}^{\cdot \cdot }$) in the HD sample strongly scatter the heat‐carrying phonons and effectively reduce the *κ*_ph_. The high‐density lattice defects such as the lattice distortions and dislocations generated during the HD process also contribute to the reduction of *κ*_ph_.^32^, ^33^, ^34^

Our recent work showcases the efficacy of intrinsic point defect engineering via tuning the Se content and the HD condition. The HD Bi_2_Te_2.3_Se_0.7_ sample attains a *zT* ≈ 1.0 at 500 K (Figure 8b). In contrast to the ZM ingot with an optimal composition *x* = 0.15–0.3,^29^, ^73^ the optimal composition of the HD sample is shifted to a significantly higher Se content *x* = 0.7 due to strong donor‐like effect.^32^ Notably, repetitive HD process further improves the *zT* of Bi_2_Te_2.3_Se_0.7_. Due to the recovery effect, the reduction of *n*_e_ leads to remarkable improvement in *α* with increasing number of times of HD. Meanwhile, the *κ*_ph_ is reduced owing to the deformation‐induced multiple‐scale defects. Consequently, a *zT* ≈ 1.2 at 445 K and an average *zT*_av_ ≈ 1.1 between 300–500 K were achieved in n‐type Bi_2_Te_2.3_Se_0.7_ hot deformed for three times (HD3), a 20% improvement over the sample hot deformed once (HD1).^32^

#### 5.1.2 p‐type *Ternary Bi_2_*_–_ *_x_Sb_x_Te_3_*

**Figure 9:**
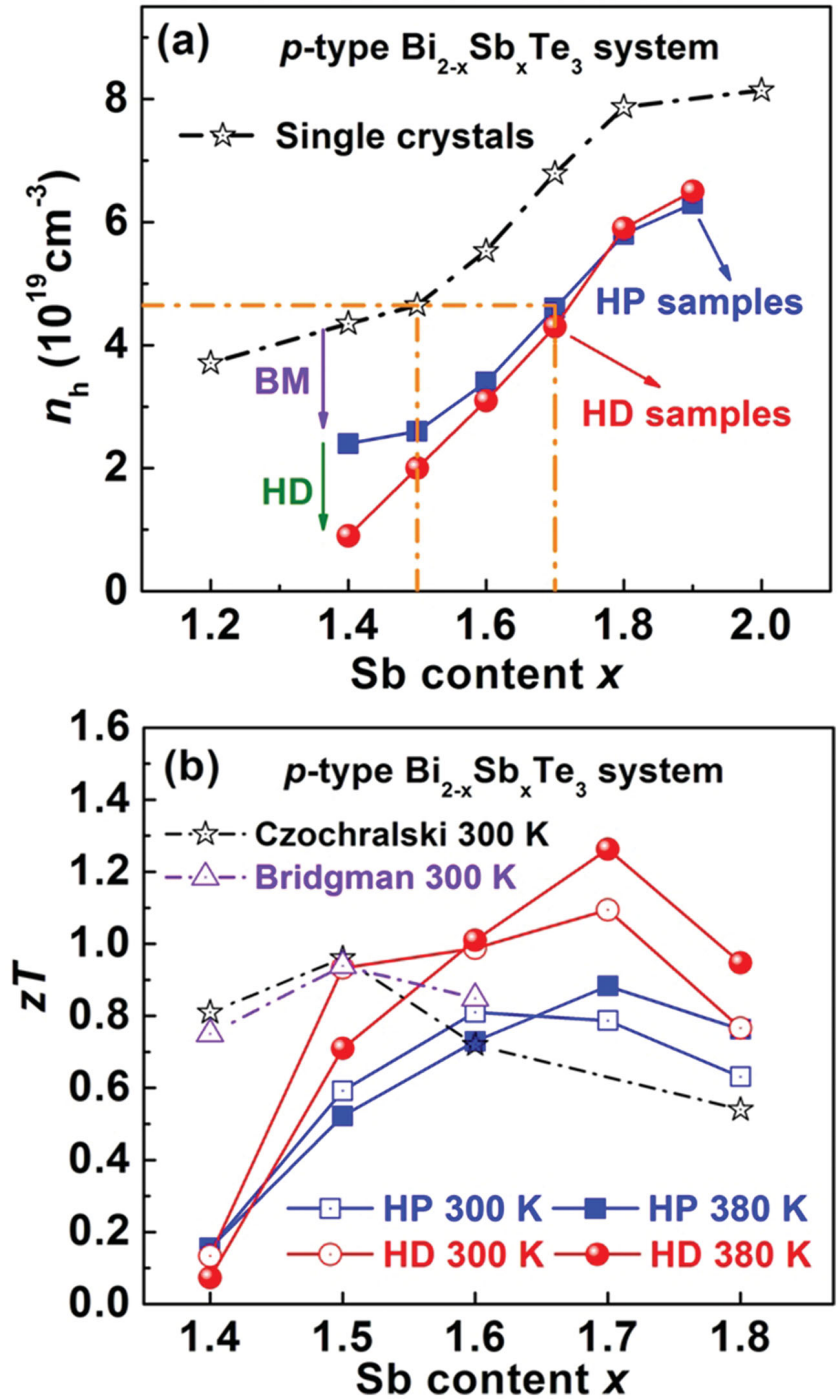
a) Room temperature carrier concentration of the undoped single crystals,^50^ HP and HD polycrystalline^32^ Bi_2–_ *_x_*Sb*_x_*Te_3_ samples. The arrows are to help visualize the variation trend of carrier concentration upon BM and HD processing. b) Sb content dependence of *zT* values for the ZM, HP, and HD Bi_2–_ *_x_*Sb*_x_*Te_3_ samples.^32^ Reproduced with permission.^32^

Intrinsic point defect engineering in p‐type Bi_2–_ *_x_*Sb*_x_*Te_3_ follows the same principle, as the underlying mechanisms are basically the same as in n‐type Bi_2_Te_3–_ *_x_*Se*_x_*. Increasing the Sb content in p‐type Bi_2−_ *_x_*Sb*_x_*Te_3_ reduces *E*_AS_ and thereby rapidly increases the *n*_h_ because of the smaller difference in *χ* and *r* between Sb and Te than that between Bi and Te (**Figure** **9**a). Compared to single crystalline Bi_0.5_Sb_1.5_Te_3_,^50^ the donor‐like defect in the BM sample partially compensates the holes and causes the reduction of *n*_h_ at all Sb contents (Figure 9a).^102^ For example, the *n*_h_ value of the BM sample with *x* = 1.7 is nearly equal to that of single crystal with *x* = 1.5.^32^ Notably, the HD process can further reduce the *n*_h_ at *x* < 1.7, while the impact of HD on the *n*_h_ is insignificant at *x* > 1.7 (Figure 9a).^32^, ^121^ We infer that at high Sb contents (*x* > 1.7) the deformation induced $V_{\mathrm{Bi}}^{\prime \prime \prime }$ (or $V\prime \prime \prime \prime_{\mathrm{Bi}}$) and $V_{\mathrm{Te}}^{\cdot \cdot }$ are depleted during the BM process, thus the donor‐like effect is less pronounced.

Compared to a value of *zT* ≈1 near room temperature in the Bi_0.5_Sb_1.5_Te_3_ ZM ingot,^29^, ^72^ the HD Bi_0.3_Sb_1.7_Te_3_ shows a higher *zT* value ≈ 1.3 at 380 K (Figure 9b).^32^ Our result is consistent with the recent work by Li et al.,^122^ in which they reported a high *zT* for the mechanical alloyed (MA) Bi_0.3_Sb_1.7_Te_3_. Notably, there is a significant improvement in the average *zT*_av_ over the temperature range studied, and the average *zT*_av_ between 300 K and 480 K for the hot deformed Bi_0.3_Sb_1.7_Te_3_ sample is 1.2. These results demonstrate again that the significance of donor‐like effect and the efficacy of intrinsic point defect engineering.

### 5.2 Tailoring Intrinsic Point Defects for Applications in Different Temperature Ranges

In this Section, we discuss how to engineer intrinsic point defects to tailor the material performance^33^, ^66^, ^94^, ^123^ in different temperature regimes.

#### 5.2.1 Room Temperature Refrigeration

**Figure 10:**
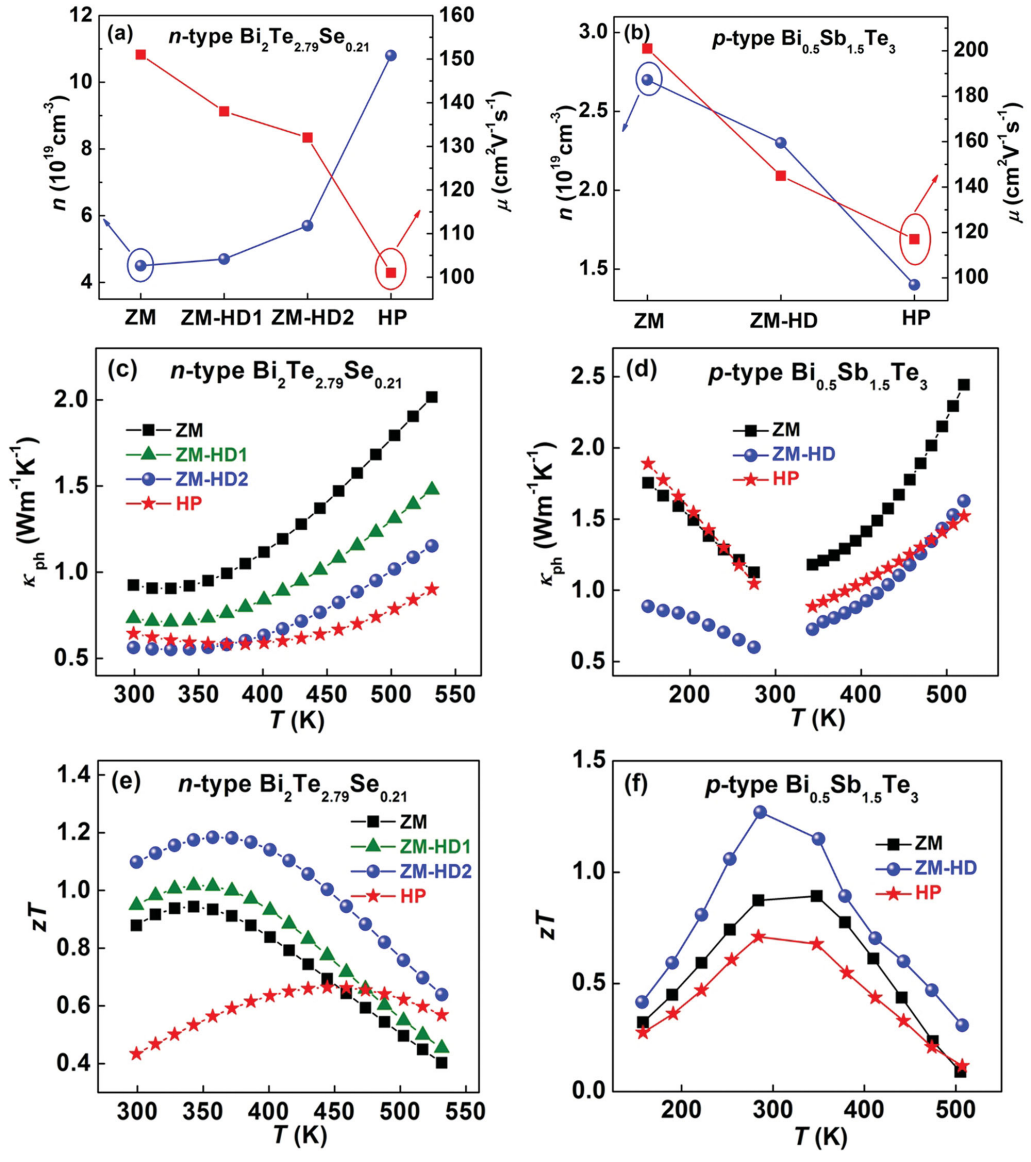
Room temperature carrier concentration and carrier mobility of a) n‐type Bi_2_Te_2.79_Se_0.21_ alloys,^33^ and b) p‐type Bi_0.5_Sb_1.5_Te_3_ alloys.^112^ Temperature dependent lattice thermal conductivity of c) n‐type Bi_2_Te_2.79_Se_0.21_ alloys,^33^ and d) p‐type Bi_0.5_Sb_1.5_Te_3_ alloys.^112^ Temperature dependence of *zT* of e) n‐type Bi_2_Te_2.79_Se_0.21_ alloys,^33^ and f) p‐type Bi_0.5_Sb_1.5_Te_3_ alloys.^112^ a,c,e) Reproduced with permission.^33^ b,d,f) Reproduced with permission.^112^ Copyright 2013, The Royal Society of Chemistry.

The best commercial TE materials for refrigeration near room temperature are ZM n‐type Bi_2_Te_3–_ *_x_*Se*_x_* (*x* = 0.15–0.3) and ZM p‐type Bi_0.5_Sb_1.5_Te_3_ ingots. We showed that hot deforming ZM ingots *without intermediate BM process* (namely, direct HD) is an effective way to enhance TE performance near room temperature.^33^, ^112^ The donor‐like effect introduced by direct HD is weaker than that with intermediate BM process because of less deformation and a stronger recovery effect.^2^, ^32^, ^33^, ^66^, ^67^ The carrier concentration slightly increases (decreases) for the n‐type ZM Bi_2_Te_2.79_Se_0.21_ (p‐type ZM Bi_0.5_Sb_1.5_Te_3_) sample upon direct HD, a high *α* value is thus retained (**Figure** **10**a,b).^33^, ^112^ In contrast, the *n*_e_ of the n‐type HP Bi_2_Te_2.79_Se_0.21_ sample is nearly tripled and the *n*_h_ of the p‐type HP Bi_0.5_Sb_1.5_Te_3_ sample is reduced nearly by half due to a stronger donor‐like effect introduced by HP.^33^

Compared with the n‐ and p‐type ZM ingots, all ZM‐HD samples exhibit somewhat lower carrier mobility *μ*, due to the weakened texture and increased grain boundary density.^33^, ^112^ As expected, the fine‐grained HP sample (subject to BM) exhibits the lowest *μ*. The HD sample shows moderately degraded *μ* compared with the HP sample owing to largely retained textures and coarse grain sizes. Importantly, the weak donor‐like effect, which leads to an increase of *σ* in n‐type sample and an increase of* α* in p‐type sample, offsets the adverse effect of *μ* degradation on the *PF* .^33^, ^112^ Since the electrical properties of the n‐type sample tend to be more sensitive to the texture and the carrier concentration variation than the p‐type sample,^33^, ^124^, ^125^ HD is recommended for the n‐type sample.

Concerning the *κ*_ph_, direct HD introduces multi‐scale microstructures, including micro scale grains and reduced texture, nanoscale distorted regions, and atomic scale line and point defects.^33^, ^112^ These multi‐scale scattering centers can effectively scatter heat‐carrying phonons with a wide wavelength range and thus effectively suppress the *κ*_ph_ (Figure 10 c,d).^33^, ^112^ As a result, the maximum *zT* reaches ≈1.2 at 357 K and ≈1.3 near room temperature for n‐type ZM‐HD2 Bi_2_Te_2.79_Se_0.21_ (HD2 denotes that the sample is hot deformed twice) and p‐type ZM‐HD Bi_0.5_Sb_1.5_Te_3_, respectively (Figure 10e,f). In comparison, the HP samples (subject to BM) show a lower *zT* than the ZM ones owing to a larger *PF* degradation than the reduction of *κ*_ph_.^33^, ^112^

#### 5.2.2 Low‐Temperature Power Generation

**Figure 11:**
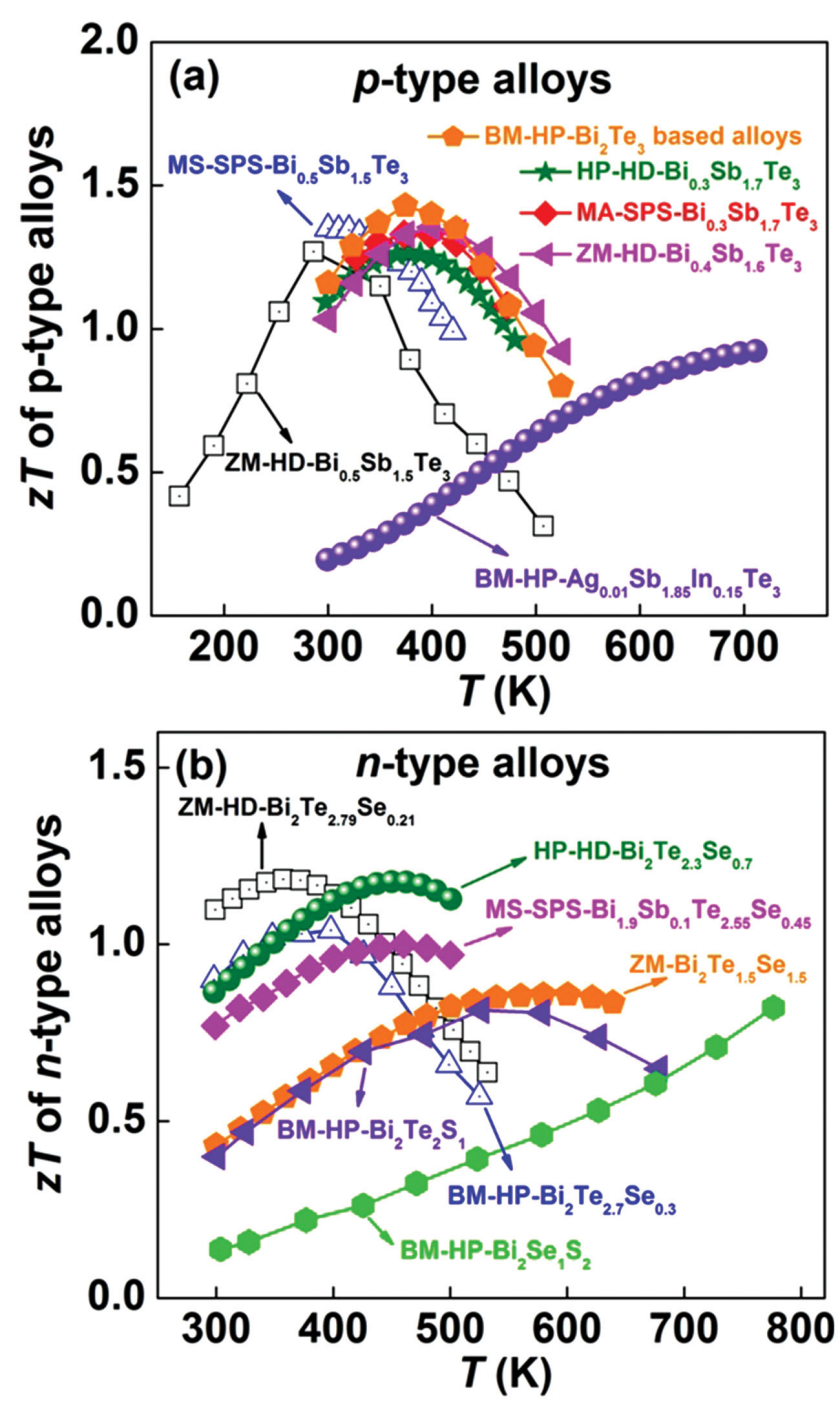
a) *zT* curves of p‐type (Bi,Sb)_2_(Te,Se)_3_ alloys.^13^, ^15^, ^66^, ^94^, ^112^, ^122^, ^126^ b) *zT* curves of n‐type (Bi,Sb)_2_(Te,Se)_3_‐based alloys.^32^, ^33^, ^39^, ^127^, ^128^

The abundant low to mid‐temperature (below 500 K) waste heat from industry sectors and automobile exhaust warrants the development of higher performance TE materials in this temperature range. However, the small band gap of n‐type Bi_2_Te_2.7_Se_0.3_ and p‐type Bi_0.5_Sb_1.5_Te_3_ inherently restricts their promise because of the detrimental ambipolar effect (i.e., the excitation of minority carriers).^66^ In addition, the maximum *zT* of p‐type Bi_2–_ *_x_*Sb*_x_*Te_3_ material needs to be shifted to higher temperatures. To this end, one can either broaden the band gap or increase the concentration of majority carriers. Notably, one can achieve both tasks via increasing the Sb content in Bi_2–_ *_x_*Sb*_x_*Te_3_ system, a high *zT* value of ≈1.3 was obtained near 380 K in HP‐HD Bi_0.3_Sb_1.7_Te_3_ (**Figure** **11**a).^66^ Li et al. also reported a *zT* value ≈1.33 at 373 K in mechanically alloyed Bi_0.3_Sb_1.7_Te_3_ with SiC nanoparticles.^122^ Compared to HP‐HD sample, less Sb (*x* = 1.6) is needed for the optimal carrier concentration in HD‐ZM sample.^126^

As mentioned above, powder metallurgy processing leads to a strong donor‐like effect and thus a high *n*_e_, making n‐type Bi_2_Te_3–_ *_x_*Se*_x_* (0 < *x* < 1) more suitable for application of low‐temperature power generation.^32^ For instance, the n‐type HD Bi_2_Te_2.3_Se_0.7_ subject to BM has a peak *zT* of 1.2 at 445 K^32^ (Figure 11b). Combining melt‐spinning (MS) and spark plasma sintering (SPS), Wang et al. reported a maximum *zT* of 1.0 at 460 K for n‐type Bi_1.9_Sb_0.1_Te_2.55_Se_0.45_.^127^ Yan et al. reported an ≈22% improvement in peak *zT* value from 0.85 to 1.04 at 398 K in n‐type Bi_2_Te_2.7_Se_0.3_ HD2 samples.^128^

#### 5.2.3 Mid‐Temperature Power Generation

Using V_2_VI_3_ compounds in mid‐temperature (above 500 K) applications demands a larger band gap *E*_g_. The binary Sb_2_Te_3_ has the largest *E*_g_ ≈ 0.20 eV among all the p‐type Bi_2–_ *_x_* Sb*_x_*Te_3_ materials.^129^ However, the binary Sb_2_Te_3_ is plagued by the presence of numerous $\mathrm{Sb}\prime_{\mathrm{Te}}$, which leads to a value of *n*_h_ ≈ 10^20^ cm^–3^ and thus a low *α* and a high *κ*_el_.^29^, ^49^ Doping by sulfur^130^ or indium^71^ can increase the *E*_AS_ of $\mathrm{Sb}\prime_{\mathrm{Te}}$ and hence reduce the *n*_h_. The *E*_g_ of Sb_2_Te_3_ can be broadened by alloying with In_2_Te_3_ (*E*_g_ ≈ 1.2 eV)^131^ or Sb_2_S_3_ (*E*_g_ ≈ 1.67 eV),^132^ thereby suppressing the detrimental bipolar effect in mid‐temperature range. As a result, a maximum *zT* ≈ 0.92 at 710 K and an average *zT* ≈ 0.8 between 500 and 710 K were obtained in (In, Ag) co‐doped Sb_2_Te_3_ (Figure 11a).^94^ Density functional theory calculations by Mehta et al. suggested that subatomic‐percent Sulfur doping of nanostructured Sb_2_Te_3_ holds the promise of *zT* ≈ 1.7 at 600 K and ≈1.6 at 800 K.^130^

In n‐type Bi_2_Te_3–_ *_x_*Se*_x_*, increasing the Se content increases the *E*_g_ and it increases the *n*_e_ in conjunction with doping by iodine. The iodine‐doped ZM Bi_2_Te_1.5_Se_1.5_ shows a maximum *zT* of 0.86 at 600 K (Figure 11b).^39^ We showed that the repetitive HD Bi_2_Te_2_Se_1_ material has a *zT* value of 1.0 at 513 K.^67^ As a comparison, single crystalline Bi_2_Te_2_Se_1_ is located right at the point of p–n transition that has the lowest *σ* and *α*,^32^ the *E*_g_ of single crystalline Bi_2_Te_3–_ *_x_* Se*_x_* happens to reach its maximum at *x* = 1.0, above which the *E*_g_ starts to decrease with increasing *x* value.^133^ To ease this restriction, the HD process and thus the donor‐like effect are utilized.^32^ Liu et al. recently conducted a systematic study of n‐type Bi_2_Te_3_–Bi_2_Se_3_–Bi_2_S_3_ system.^134^ These results showed that Bi_2_Te_2_S_1_ has a peak *zT* value ≈ 0.8 at 573 K and Bi_2_Se_1_S_2_ ≈ 0.8 at 773 K upon high energy BM followed by the HP process. It is plausible to infer that the donor‐like effect plays a key role in these materials.

## 6 Approaches beyond Intrinsic Point Defect Engineering

The focus of Section 6 is on the underheeded role of intrinsic point defects in the (i) nanostructuring approach and (ii) texturing approach. Extensively employed in V_2_VI_3_ materials but *without* explicitly containing “intrinsic point defects” in their names, the nanostructuring and texturing approach involve powder metallurgy processes such as BM, HD, HP. These processes are the same ones we employ to create intrinsic point defects (cf. Section 3.3 and 3.4). *Hence the proper assessment of nanostructuring and texturing approach is subject to a proper assessment of donor‐like effect and recovery effect*.

While the nanostructuring approach was initially proposed to enhance the electrical properties of TE material via quantum confinements,^135^, ^136^ most advances in enhancing *zT* are attained by the reduction of *κ*_ph_ in nanostructured TE material. On one hand, the nanostructuring process introduces numerous grain boundaries that strongly scatter heat‐carrying phonons. On the other hand, it is risky to assert that grain boundary scattering is the primary mechanism underlying the reduction of *κ*_ph_. A good example is the reduction of *κ*_ph_ in Bi_2_Te_3_ nanocrystals (cf. Section 4.2),^120^ in which the deformation‐induced vacancies dominate over grain boundaries.

Nanostructuring approach can be categorized into two basic classes: bottom‐up and top‐down. In a typical bottom‐up approach, nanostructures are firstly prepared by BM,^137^, ^138^, ^139^ or MA^140^, ^141^, ^142^, ^143^ before consolidation to yield nanostructured bulk materials. A high *zT* value of 1.4 using ZM ingots as the feedstock^13^ and a high *zT* value of 1.3 using elemental chunks as the feedstock^138^ were attained in p‐type Bi_2−_ *_x_*Sb*_x_*Te_3_ nanocomposites by a high‐energy‐BM‐HP procedure. In comparison, HD is an effective top‐down approach for creating nanostructures and enhancing the *zT* of both p‐ and n‐type (Bi,Sb)_2_(Te,Se)_3_‐based materials. The significant reduction in *κ*_ph_ of HD‐ZM sample is ascribed to effective phonon scattering by multi‐scale microstructures.^33^

Texture refers to the misorientation between grains. To the first order approximation, texture is independent of intrinsic point defects. Texture is found to be crucial for the carrier mobility *μ*,^144^, ^145^, ^146^, ^147^, ^148^, ^149^, ^150^, ^151^ intrinsic point defects are shown to affect the carrier concentration *n* (cf. Section 2 and 3) while they both control the anisotropy of {*σ*, *α*, *κ*}. The commercial V_2_VI_3_ TE materials are fabricated by unidirectional crystal growth methods such as Bridgman,^152^ Czochralsky,^153^ and zone‐melting (ZM)^154^ technique, which lead to textures in the as‐grown ingots. Advanced powder metallurgy methods, including HP,^155^ SPS,^156^ hot extrusion,^157^, ^158^, ^159^, ^160^, ^161^, ^162^ shear extrusion,^163^, ^164^ powder extrusion,^165^ and equal channel angular extrusion^166^, ^167^ have been utilized to introduce textures in V_2_VI_3_ materials. It is plausible to assume that these deformation processes involves the donor‐like effect (cf. Section 3.3). For example, Zhao et al. prepared fine‐grained n‐type Bi_2_Te_3_ materials with preferred grain orientation by using SPS as a hot forging tool.^104^ We have employed HD process to obtain high performance p‐ and n‐type V_2_VI_3_ materials.^2^, ^32^, ^66^, ^67^ The degree of texture can be controlled by the HD temperature,^2^ the number of times of HD,^67^ and also the deformation strain.^66^

Notably, the carrier concentration *n* strongly affects the anisotropy of {*σ*, *α*, *κ*}.^124^, ^125^ Increasing the *n* deforms the Fermi surface topology, making it more prolate and warped from an ellipsoidal shape. As a result, the anisotropy ratio in both *σ* and *κ* increases with increasing *n* given the same degree of texture.^124^, ^125^ As for the *α*, it is nearly isotropic in the extrinsic region,^168^ and highly anisotropic in the intrinsic region.^169^, ^170^ The *α* anisotropy is attributed to the presence of minority carriers and the difference in the ratio of hole to electron mobility along the two principal directions.^169^ Hence a synergistic implementation of texture and intrinsic point defects would help simultaneously attain an optimal *μ* and an optimized anisotropy of {*σ*, *α*, *κ*}.

## 7 Conclusions

Defects, ubiquitous and often wrongly conceived as performance limiters, are the key performance enhancer in diverse functional materials upon proper implementation. This review focuses on the underexplored intrinsic point defects (i.e., vacancies and antisite defects) in V_2_VI_3_ semiconductors and their derivatives, regarding the compositional, mechanical and thermal control as well as their interplay with other defects towards higher thermoelectric performance. It is not our aim to emphasize the significance of intrinsic point defects over other types of defects; rather, we intend to clarify the causal chain in the synthesis‐structure‐property correlation. We summarized our understanding of intrinsic point defects in a (*χ, r*)‐model and discussed the donor‐like effect and the recovery effect in V_2_VI_3_ compounds.

The study of intrinsic point defects in V_2_VI_3_ compounds is not yet complete, especially regarding the role of intrinsic point defects in nanostructuring and texturing approaches (cf. Section 6), which warrants further investigations. Nonetheless, the new insights derived herein open a promising avenue for further improving the thermoelectric performance of other compounds and, in a wider context, contribute to the development of advanced functional materials by rational defect design in the long run.
